# Fast Automatic Registration of UAV Images via Bidirectional Matching

**DOI:** 10.3390/s23208566

**Published:** 2023-10-18

**Authors:** Xin Luo, Zuqi Wei, Yuwei Jin, Xiao Wang, Peng Lin, Xufeng Wei, Wenjian Zhou

**Affiliations:** 1 Yangtze Delta Region Institute (HuZhou), University of Electronic Science and Technology of China, Huzhou 313099, China; luoxin@uestc.edu.cn (X.L.); wangxiao29@std.uestc.edu.cn (X.W.); 2019070403014@std.uestc.edu.cn (P.L.); xufengw@std.uestc.edu.cn (X.W.); 2 School of Electrical and Information Engineering, Panzhihua University, Panzhihua 617000, China; jinyuwei@alu.uestc.edu.cn; 3 School of Automation Engineering, University of Electronic Science and Technology of China, Chengdu 611731, China

**Keywords:** UAV, image registration, ORB, point feature, bidirectional matching

## Abstract

Image registration plays a vital role in the mosaic process of multiple UAV (Unmanned Aerial Vehicle) images acquired from different spatial positions of the same scene. Aimed at the problem that many fast registration methods cannot provide both high speed and accuracy simultaneously for UAV visible light images, this work proposes a novel registration framework based on a popular baseline registration algorithm, ORB—the Oriented FAST (Features from Accelerated Segment Test) and Rotated BRIEF (Binary Robust Independent Elemental Features) algorithm. First, the ORB algorithm is utilized to extract image feature points fast. On this basis, two bidirectional matching strategies are presented to match obtained feature points. Then, the PROSRC (Progressive Sample Consensus) algorithm is applied to remove false matches. Finally, the experiments are carried out on UAV image pairs about different scenes including urban, road, building, farmland, and forest. Compared with the original version and other state-of-the-art registration methods, the bi-matching ORB algorithm exhibits higher accuracy and faster speed without any training or prior knowledge. Meanwhile, its complexity is quite low for on-board realization.

## 1. Introduction

Because of its flexibility, high efficiency, and low cost, UAV (Unmanned Aerial Vehicle) remote sensing technology has gradually emerged in many fields, such as accurate agriculture, resources investigation, environment management, and disaster monitoring [1,2,3]. How to yield high-precision registered UAV images quickly has become an inevitable challenge to the wide application of UAV technology [4,5,6,7,8,9]. However, the high resolutions of UAV images have a great influence on the detection and matching of image feature points. Meanwhile, there is a series of parameters in collecting UAV images, such as flight speed, flight height, and weather conditions, which make detection and matching of image feature points more difficult to achieve. Hence, the automatic registration of UAV remote sensing images is worth studying comprehensively.

Recently, the issue of image registration has received considerable intensive attention from scholars. For instance, the SIFT (Scale Invariant Feature Transform) operator can obtain a sort of feature invariant to image resolution, rotation, and scaling [10]. However, the SIFT algorithm will yield 128-dimensional features, which is heavily time-consuming. Hence, the feature dimension in the SURF (Speeded-Up Robust Features) algorithm is reduced to 64 through wavelet transform, which efficiently enhances the registration speed [11,12]. Moreover, focused on matching different features, Zhang et al. combined the image enhancement technology with the SURF algorithm to obtain better feature points and matching efficiencies [13]. Wei et al. divided an image into super-pixels to calculate the information entropy of each region [14]. The redundancies in feature points are diminished according to the values of their information entropy. Zhang et al. proposed a threshold evaluation strategy to extract SURF rough matching points and employed the RANSAC (Random Sample Consensus) algorithm to eliminate gross errors [15]. Furthermore, in order to constrain the regions for fine matching, a similarity function of Delaunay triangles constructed by Harris points is introduced to obtain matched triangle pairs. At present, ORB (the Oriented FAST and Rotated BRIEF) is a fast local feature detection operator [16], which is improved based on the FAST (Features from Accelerated Segment Test) [17] and BRIEF (Binary Robust Independent Elemental Features) algorithm [18]. It overcomes the lack of scale and rotation invariances in FAST and is faster than most classical image registration algorithms. However, since it has the disadvantage of low accuracy, this work attempts to improve the comprehensive performances of ORB on UAV images. The main contributions of this work are presented below.

- The ORB operator is utilized to extract image feature points since many fast registration methods cannot simultaneously provide high speed and accuracy for UAV images. Its complexity is quite low for on-board realization.
- The KNN-based search and the Hamming distance are used in the initial matching of feature points; two bi-directional matching strategies are designed for their fine matching; and the progressive consistency algorithm (PROSAC) is exploited to remove false matches and fit a geometric transformation model to enhance registration accuracy.
- The validation experiments were carried out on the UAV images from five typical scenes, which were acquired by different UAV image sensors. The results indicate that the proposed algorithm can achieve higher accuracy and faster speed than some existing fast registration algorithms.

The remainder of this article is organized as follows. Section 2 describes an improved ORB registration method. After images are down-sampled to speed up feature matching, an improved ORB operator and a bi-directional matching strategy are utilized to detect and match feature points in UAV images. After the initial matching, feature point pairs are obtained, and the PROSRC (Progressive Sample Consensus) algorithm is performed to screen out mismatched points to obtain an accurately matched point-pair set and final transform parameters [19]. In Section 3, our proposed algorithm is compared with some existing registration methods using UAV images acquired from different scenarios and sensors. The conclusion and discussion are presented in Section 4.

## 2. Methodology

**Figure 1:**
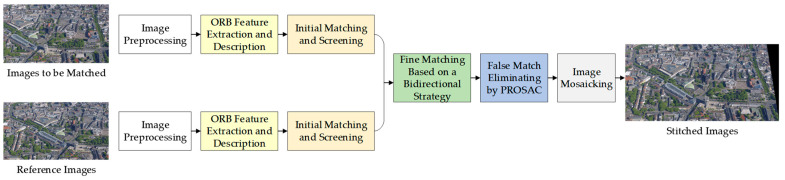
The flow chart of the proposed fast registration algorithm for UAV (Unmanned Aerial Vehicle) images.

Because of their high resolutions, it usually takes a long time for common image registration algorithms to process UAV images. However, there are often real-time requirements in many applications of UAV images. Therefore, based on an improved ORB operator, a bi-directional matching strategy is combined with PROSAC to pair extracted feature points, which can enhance the accuracies of the proposed registration algorithm. The registration procedure of our algorithm is illustrated in Figure 1.

### 2.1. Image Preprocessing

#### 2.1.1. Down-Sampling

On account of the high resolutions of UAV images, an appropriate scaling factor is chosen to adjust the sizes of input images. If the initial size of an image is *M × N*, the width and height of the image are reduced to 1/*n* of the original one after being down-sampled by *n*. That is, the size of the obtained image becomes (*M × N*)/*n*, where the pixel values of each *n × n* region in the original image are replaced by the mean pixel value of the region. This operation can reduce the speeds of image loading and processing, which is beneficial to the real-time performances of registration algorithms.

#### 2.1.2. Graying

**Figure 2:**
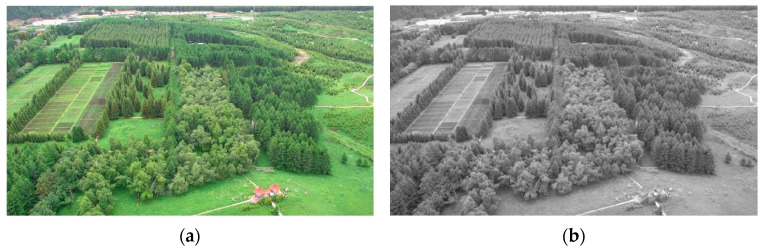
A graying instance of UAV images: (**a**) a visible image; (**b**) the grayed image.

It is known that the structure and texture characteristics of visible images usually will not vary with optical bands. For the sake of processing speeds, grayscale images are used as the basis of image registration in this work, and input RGB images are grayed according to the importance of three optical bands. The intensity of each pixel is generated from a weighted average of three different components. A graying instance of UAV images is given in Figure 2. Considering the sensitivity of human eyes to color information, the intensity of an arbitrary pixel can be denoted as

(1) f(i,j)=0.30R(i,j)+0.59G(i,j)+0.11B(i,j)

where *R*(*i*, *j*), *G*(*i*, *j*), and *B*(*i*, *j*) represent the three components at an arbitrary piont (*i*, *j*) in an input image [20].

### 2.2. ORB Brief

To ensure scale invariances, a Gaussian pyramid and patch centroid calculation based on FAST are combined in ORB. Moreover, an improvement on the BRIEF algorithm for rotation invariance is designed for constructing feature descriptors of ORB [21,22,23].

#### 2.2.1. Feature Point Extraction

In terms of feature point detection, the kernel method of ORB is based on FAST [24,25,26]. If the difference in the gray level between the central pixel and one of its neighbors is significant enough, the point will be regarded as a feature point. This detection method of feature points not only preserves image features as much as possible but also greatly reduces computational complexity. It will bring about a significant improvement in the speed of the ORB algorithm. For an arbitrary detected feature point, the moment of its neighbor patch *m_pq_* is defined as

(2) $$m_{pq}=\sum_{x,y}x^{p}y^{q}I(x,y),\begin{array}{cc} & p,q\in \{0,1\} \end{array}$$

where *I*(*x*, *y*) is the intensity of the pixel at (*x*, *y*). Then, the centroid coordinate *C* can be determined by

(3) $$C=\left(\frac{m_{10}}{m_{00}},\frac{m_{01}}{m_{00}}\right)$$

Suppose that the feature point is *O*. The angle of the vector $\vec{OC}$ *θ* is regarded as the orientation of the feature point, as given below.

(4) $$\theta =\arctan2\left(\frac{m_{01}}{m_{10}}\right)$$

where arctan2 is the quadrant-aware arctan [24].

#### 2.2.2. Feature Descriptor Construction

After the feature points in images are found, an improved BRIEF algorithm, rBRIEF (rotation-aware BRIEF), is adopted in ORB to describe the features of feature points [27,28,29]. Binary coding is employed by BRIEF to construct feature description vectors, which is very helpful for accelerating calculation. Since BRIEF does not possess rotation invariance, the specific improvements on the BRIEF algorithm in ORB are as follows [24].

Give an *M* × *M* pixel smoothed patch *P*. A binary testing *τ*(*P*; *u*, *v*) can be defined as

(5) $$\tau (P;u,v)=\left\{ \begin{array}{l} 1\begin{array}{cc} & P(u)<P(v) \end{array}\\ 0\begin{array}{cc} & otherwise \end{array} \end{array}\right.$$

where *P*(*u*) and *P*(*v*) are the intensities of the pixels at two points *u* and *v*, respectively. For *n* pairs of points around a feature point, the generated feature descriptor *f_n_*(*P*) is an *n*-dimensional binary string, as follows.

(6) $$fn(P)=\sum_{1\leq i\leq n}2^{i-1}\tau (P;u_{i},v_{i})$$

According to the orientation of feature points, a corresponding matrix ***S*** is constructed as given in the following Equation.

(7) $$S=\left[\begin{array}{c} u_{1},u_{2},\cdots ,u_{n}\\ v_{1},v_{2},\cdots ,v_{n} \end{array}\right]$$

Since the orientation of the feature point is *θ*, the corresponding rotation matrix ***R****_θ_* is defined by

(8) $$R_{\theta }=\left[\begin{array}{cc} \cos\theta & \sin\theta \\ -\sin\theta & \cos\theta \end{array}\right]$$

Then, if the matrix ***S*** is transformed by the rotation matrix ***R****_θ_*, a new feature description matrix ***S****_θ_* is obtained, which can be derived as below.

(9) $$S_{\theta }=R_{\theta }S=\left[\begin{array}{cc} \cos\theta & \sin\theta \\ -\sin\theta & \cos\theta \end{array}\right]\left[\begin{array}{c} u_{1},u_{2},\cdots ,u_{n}\\ v_{1},v_{2},\cdots ,v_{n} \end{array}\right]$$

Consequently, the improved descriptor *g_n_*(*P*, *θ*) can be expressed as

(10) $$g_{n}(P,\theta )=f_{n}(P)|(u_{i},v_{i})\in S_{\theta }$$

### 2.3. Initial Matching and Screening

The initial matched point pairs are yielded by comparing the extracted feature points in the image to be registered and its reference image. In practice, the comparison of image point features is realized based on the similarities of feature points. The Hamming distance is chosen to describe the similarities between different points in this research [30,31,32]. To enhance the efficiency of initial matching, a fast search based on the K-Nearest Neighbor (KNN) algorithm is adopted in this work [33,34]. Unlike common violent matching methods, it searches for a corresponding point with the highest similarity for a point to be registered in a determined region of the reference image. Their Hamming distances to the point to be registered are calculated. Two matched points, optimal and suboptimal, are found by KNN. Lowe’s algorithm is adopted to compare whether the ratio of the two distances is less than a given threshold [35]. If so, the current matching relationship will be regarded as an acceptable matched point pair. Otherwise, the matching relationship will be eliminated.

### 2.4. Bi-Directional Fine Matching

**Figure 3:**
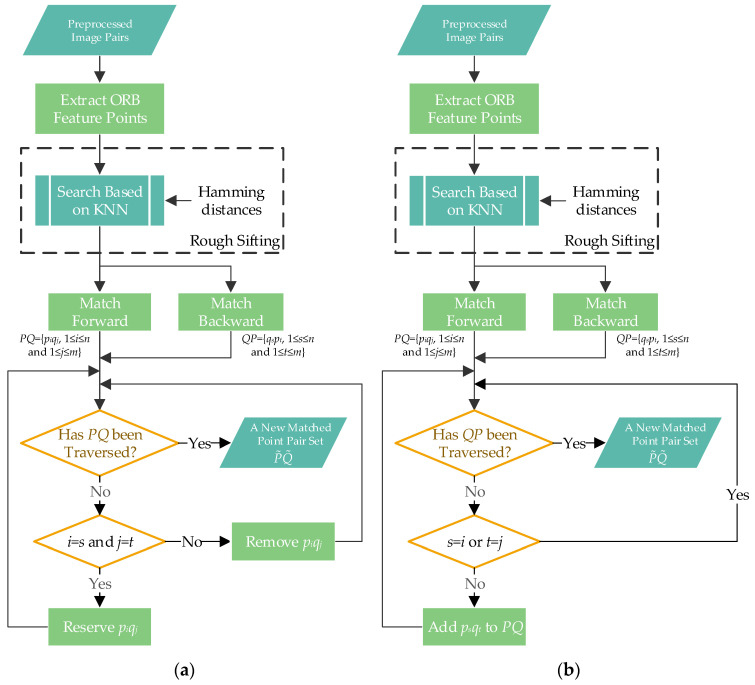
The flow chart of bi-directionally matching UAV images: (**a**) Strategy 1; (**b**) Strategy 2.

The specific means of feature point detection and feature vector construction applied in ORB significantly contribute to processing speeds. Still, the algorithm’s performances are poor in terms of accuracy. Hence, it is necessary to accomplish the fine matching of feature points through an appropriate strategy. However, the matching procedures of the most common registration methods are unidirectional, which only search matching objects for feature points in reference images. There are many errors and omissions in their matching results. Focused on these problems, two bi-directional matching strategies are proposed in this work, as depicted in Figure 3.

Strategy 1: The primary idea of the first bi-directional matching strategy is to remove wrongly matched points in initial matching results. As presented in Figure 3a, its specific procedure is as follows. Suppose that the feature points detected in an image to be registered are marked as {*p*_1_, *p*_2_, …, *p_n_*}, and the feature points detected in its reference image are marked as {*q*_1_, *q*_2_, …, *q_m_*}. After forward matching, a set of matched feature points in the two images is obtained and denoted as *PQ* = {*p_i_q_j_*, 1 ≤ *I* ≤ *n* and 1 ≤ *j* ≤ *m*}. Similarly, after backward matching, a set of matched feature points in the two images can be represented by *QP =* {*q_s_p_t_*, 1 ≤ *s* ≤ *n* and 1 ≤ *t* ≤ *m*}. Then, the backward matching results are traversed according to the forward matching result. If it is found that there are two identical pairs of matched points, i.e., *p_i_q_j_* and *q_t_p_s_* with *i* = *s* and *j* = *t*, the point pair *p_i_q_j_* (or *q_t_p_s_*) can be regarded as a correct matching relationship and will be preserved. Otherwise, the matched point pairs in the backward matching result will be eliminated. Thereby, a new set of matched feature points $\tilde{P}\tilde{Q}$ can be built more reliably through this bi-directional matching strategy.

Strategy 2: The other bi-directional matching strategy is to compensate missed feature points into unidirectional results. The concrete realization is elaborated as follows. If there is a matched point pair *q_s_p_t_* (1 ≤ *s* ≤ *n* and 1 ≤ *t* ≤ *m*) in the backward matching result, but *q_s_* and *p_t_* do not appear in the forward matching result *PQ*, *q_s_p_t_* will be deemed as a matching pair missed in forward matching. Accordingly, the point pair *p_t_q_s_* will be added to the set *PQ*. Then, a new set $\tilde{P}\tilde{Q}$ containing more matched feature points is obtained until all the point pairs in the backward matching result are examined. The detail matching flow is elaborated in Figure 3b.

### 2.5. False Match Elimination and Transform Model Fitting

In this work, PROSAC is employed to remove mismatched feature points further from bi-directional rough matching results [36]. It is known that the classical RANSAC method is realized by random sampling of rough matching results to fit transform models. This method is random and heavily depends on pre-set iteration times for fitting models. Nevertheless, PROSAC is constructed by sampling an increasing set of optimally matched point pairs, which have been ranked based on a certain similarity measure. Sampling and fitting models based on top-ranked point pairs help enhance the success rates of obtaining correct models. Meanwhile, the randomness of the algorithm decreases obviously. Specifically, matched point pairs in $\tilde{P}\tilde{Q}$ are sorted from near to far according to Hamming distances of feature descriptors. Then, four point pairs are randomly chosen from the top *m* pairs in the reordered feature point-pair set, which can be used to fit a 3 × 3 homography matrix ***H***, which can satisfy the following condition.

(11) $$\left[\begin{array}{c} x\prime \\ y\prime \\ 1 \end{array}\right]∼H_{3\times 3}\left[\begin{array}{c} x\\ y\\ 1 \end{array}\right]$$

where (*x*, *y*) and (*x*’, *y*’) correspond to point coordinates in the image to be registered and the reference image, respectively. According to the current parameters of the transform matrix ***H***, the coordinates of all other points in the image to be registered are transformed by ***H*** into the coordinate system of the reference image. Then, these points are classified into inner or outlier points according to the differences between their projection coordinates and corresponding point coordinates in the reference image. These inner points are refined matched point pairs, and ***H*** will be renewed in light of them. Repeatedly, the inner point set and ***H*** will be updated until the pre-set times of iterations are reached.

## 3. Results

The experimental hardware platform includes an Intel Core i5-4590K processor with a main frequency of 3.5 GHz and 16 GB RAM. The experimental software environment contains a 64-bit operating system Windows 10, a programming tool Visual Studio 2017, and an open-source library OpenCV 3.20. The comprehensive performances of the proposed registration algorithm are verified in the experiments by using UAV images of different sensors acquired from different scenes, including urban, road, building, farmland, and forest. Moreover, the improved ORB algorithm is compared with the existing popular algorithms, including SIFT [37], SURF [38], KAZE (a Japanese word) [39], AKAZE (Accelerated KAZE) [40], and its original version in terms of speed and accuracy.

### 3.1. Determining the Number of Feature Points

**Figure 4:**
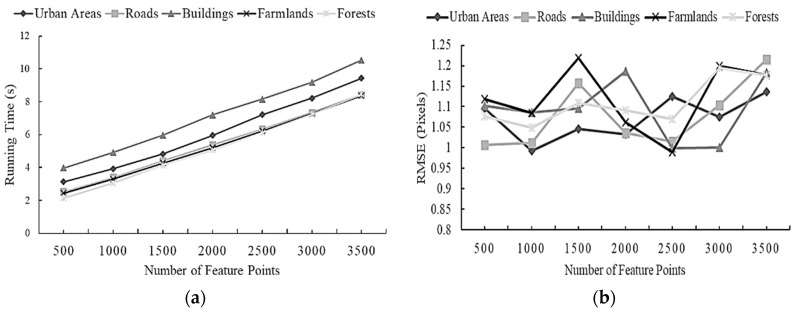
The relationship between the number of feature points and running time or RMSE (Root Mean Square Error) for five scenes: (**a**) the number of feature points vs. running time; (**b**) the number of feature points vs. RMSE.

Since the number of feature points extracted by the ORB operator is optional, a comparative experiment is carried out to determine an appropriate number of feature points for optimal registration effects. Hence, the UAV images of five scenes are registered through the different numbers of feature points extracted by the original ORB algorithm. The relationship between the number of feature points and running time or Root Mean Square Error (*RMSE*) are separately plotted in Figure 4a,b. *RMSE* can be denoted as follows [2].

(12) $$RMSE=\sqrt{\frac{1}{n}\sum_{i=1}^{n}{\left(\left\|T\left(y_{i},\theta \right)-x_{i}\right\|\right)}^{2}}$$

where ***x****_i_* and ***y****_i_* (for *i* = 1, 2, …, *n*) stand for one of the *n* matching point pairs from the image to be registered and the reference image. *T* is a transform model, and ***θ*** is the model parameter vector. ∥• is the Euclidean distance between two points. Generally, the smaller the value of RMSE, the higher the registration accuracy. From Figure 4a, it can be found that the running time of the algorithm is directly proportional to the number of feature points. Meanwhile, it can be seen from Figure 4b that there exist minimum RMSE values individually at 1000, 2000, and 2500, and most of the values of RMSE for the five senses reach the lowest when the number of feature points is 1000. Thereby, considering running time and registration accuracy, the number of feature points to be extracted is uniquely set at 1000 for different scenes in the subsequent registration experiments.

### 3.2. Registration of Scene Images

**Figure 5:**
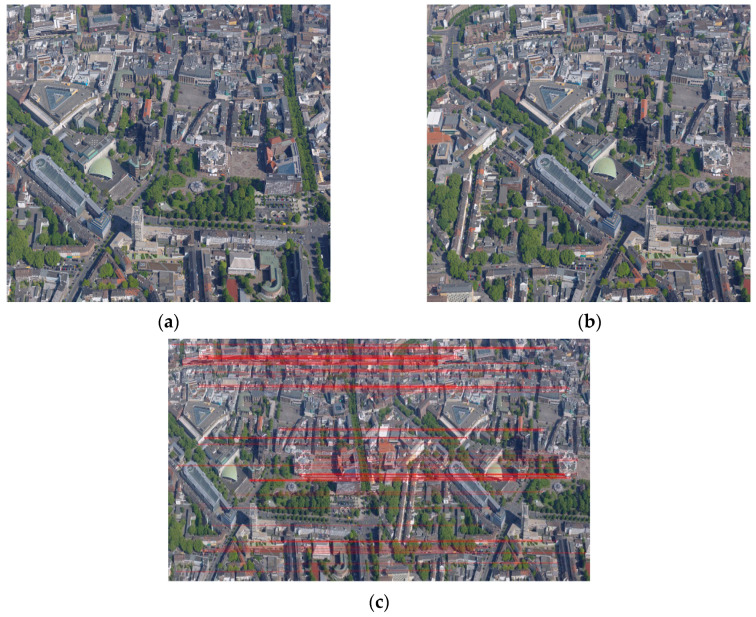
A UAV image pair from an urban scene and the feature points after the initial screening: (**a**) the image to be registered; (**b**) the reference image; (**c**) the image pair after the rough screening. The corresponding points are connected by red lines.

The sources and parameters of UAV images used in the registration experiments are listed in Table 1. The urban and building scene images are, respectively, selected from the high-altitude and near-ground drone data of an ISPRS benchmark for multi-platform photogrammetry [41]. The image pairs of a road scene are the test images provided by Pix4Dmapper v.2.0 software. The farmland and forest scene images are self-collected. Particularly, the image pair of a farmland scene was acquired by the Parrot Sequoia multispectral camera with four bands, including green, red, red-edge, and near-infrared. Its red edge images are employed as blue components in the registration of this work. The two images of each scene image-pair were taken at different angles with an overlap. The image pair of the urban scene possesses many details and is quite complex, as shown in Figure 5. As mentioned before, 1000 pairs of feature points are extracted in the two images, and only 99 pairs of points are left after initial screening, as displayed in Figure 5c.

**Figure 6:**
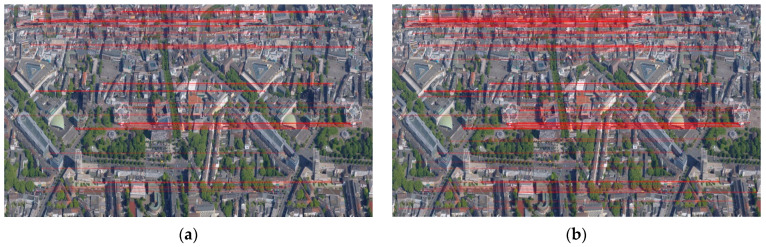
The urban image pairs after bi-directional matching according to two strategies: (**a**) Strategy 1; (**b**) Strategy 2. The corresponding points are connected by red lines.

Based on the result of rough matching, two bi-directional matching strategies are adopted in fine matching, as shown in Figure 6. There are 60 matched point pairs preserved after screening out 39 point pairs using the first bi-directional matching strategy. After processing by the second bidirectional matching strategy, 121 matching point pairs remain, including 22 supplemental point pairs.

**Figure 7:**
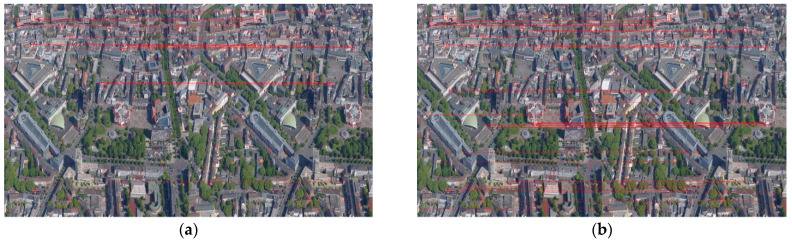
The results of bi-directional matching after eliminating false matches: (**a**) Strategy 1; (**b**) Strategy 2. The corresponding points are connected by red lines.

**Figure 8:**
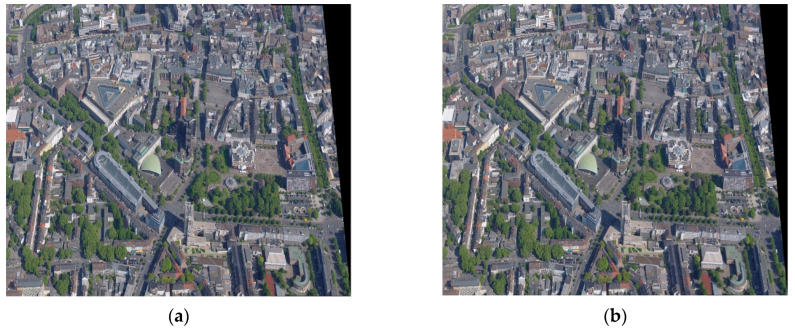
The stitched image of the urban scene by the proposed registration algorithms: (**a**) Strategy 1; (**b**) Strategy 2.

Then, the elimination results of false matches using the PROASC algorithm are shown in Figure 7, and the final registration results are presented in Figure 8. Since some pixel points in transformed images cannot be assigned directly to new grid coordinates, bi-linear interpolation is utilized to generate stitched images [42]. For the result of the first bi-directional matching strategy, 23 pairs of matched point pairs remain, and 37 pairs of matching point pairs are removed by the PROSAC algorithm. In this work, the running time and RMSE of the registration algorithm are chosen as the main indicators in performance comparisons. The running time of the registration process is 3.922 s, and the RMSE is 0.9926 pixels. The obtained transform matrix is as follows.

(13) $$H=\left[\begin{array}{ccc} 1.000727295875549 & 0.000115774389996 & -675.5347900390625\\ 0.000406843028031 & 1.000958800315857 & 118.6014556884766\\ 0.000000566664652 & 0.000000197085213 & 1 \end{array}\right]$$

For the result of the second bi-directional matching strategy, 45 pairs of matching point pairs are left, and 76 pairs of matching point pairs are removed by the PROSAC algorithm. The registration time is 4.031 s, and the RMSE is 1.0750 pixels. The corresponding perspective matrix is denoted as

(14) $$H=\left[\begin{array}{ccc} 1.000828385353088 & 0.000052663752680 & -675.6309814453125\\ 0.000271332741249 & 1.001049280166626 & 118.7060470581055\\ 0.000000538613107 & 0.000000276266348 & 1 \end{array}\right]$$

The registration accuracies of different registration algorithms for different scenes are listed in Table 2. For the five scene images, it can be seen that regarding accuracies, the proposed registration algorithms can provide obvious improvements on the original ORB algorithm, and the method based on the first bi-directional matching strategy exceeds all the other algorithms. For the scene images of urban areas, buildings, and farmlands, our algorithm even can achieve sub-pixel registration accuracies. In addition, it is revealed that the accuracy improvements of the proposed methods are related to the scenes of the experimental images. If there are rich details in images such as those found in urban, road, and building images, the accuracies can increase by more than 20.0% compared to the original ORB algorithm. If the images have fewer details, such as farmland and forest images, the accuracy enhancements are not outstanding, but still about 10.0%. The reason may be that the more details in images, the greater the differences in extracted feature points. Furthermore, the qualities of matched feature point pairs can be improved greatly after coarse and fine screening so that registration accuracies increase.

The time consumption comparison of different registration algorithms is presented in Table 2. As can be seen, the ORB algorithm has obvious advantages in running time over the other five algorithms. The reason may be that the high resolutions of these experimental UAV images result in more feature points being detected by the other five algorithms. Consequently, it takes a lot of time for these methods, especially for the KAZE algorithm, to detect and match feature points. However, it seems rational that the matching accuracy of the ORB algorithm is the lowest in Table 3 because its performance is closely related to the number of feature points. Our proposed registration methods cannot only inherit the advantage in speeds from the ORB algorithm but also achieve excellent registration qualities by combining the bi-directional matching strategies and a false-match elimination method. Hence, it can be drawn that our proposed registration algorithm realizes accuracy enhancement at the small cost of processing speeds.

**Figure 9:**
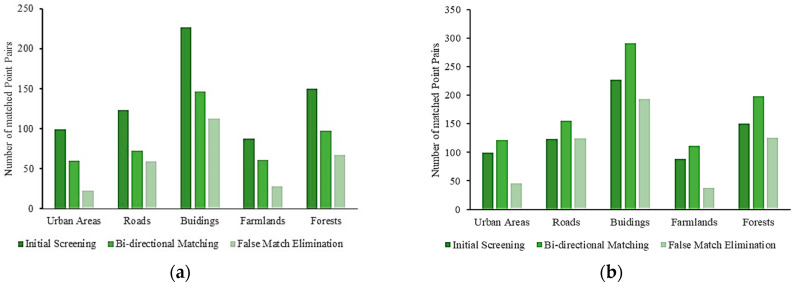
The numbers of matched point pairs in registration processes: (**a**) the first bi-directional strategy; (**b**) the second bi-directional strategy.

In addition, the numbers of matched point pairs in the five scene images after different process stages are illustrated in Figure 9. It is known that there are abundant detail features in the UAV images of urban areas, buildings, and roads. In contrast, the contents of the UAV images of farmlands and forests are relatively simple, and their feature textures are not enough. In general, a sufficient number of feature points need to be extracted by matching methods effectively for fitting transformation model parameters. For the scene images of urban areas, buildings, and roads, a relatively large number of representative feature points can be detected. Although it benefits the increases in registration accuracies, it also spends more time in additional calculations. For the images of farmlands and forests, fewer and lower representative feature points are detected, which leads to lower registration accuracies but less calculation time.

**Figure 10:**
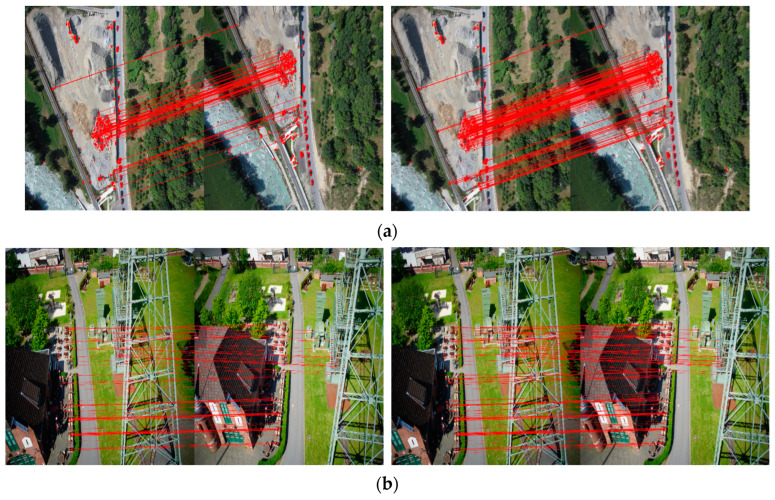

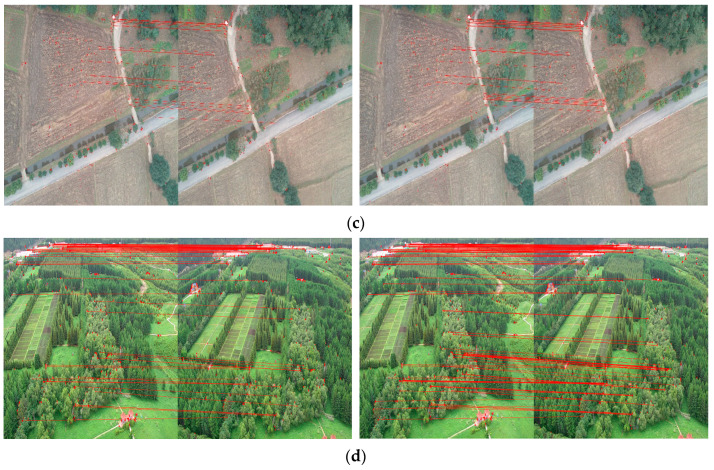
The different image pairs after bi-directional matching according to two strategies: (**a**) roads; (**b**) buildings; (**c**) farmlands; (**d**) forests. The corresponding points are connected by red lines.

**Figure 11:**
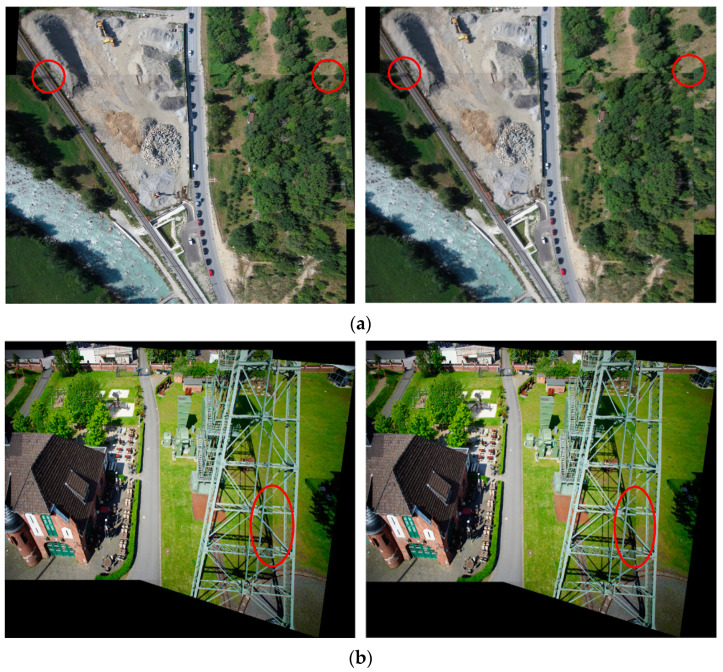

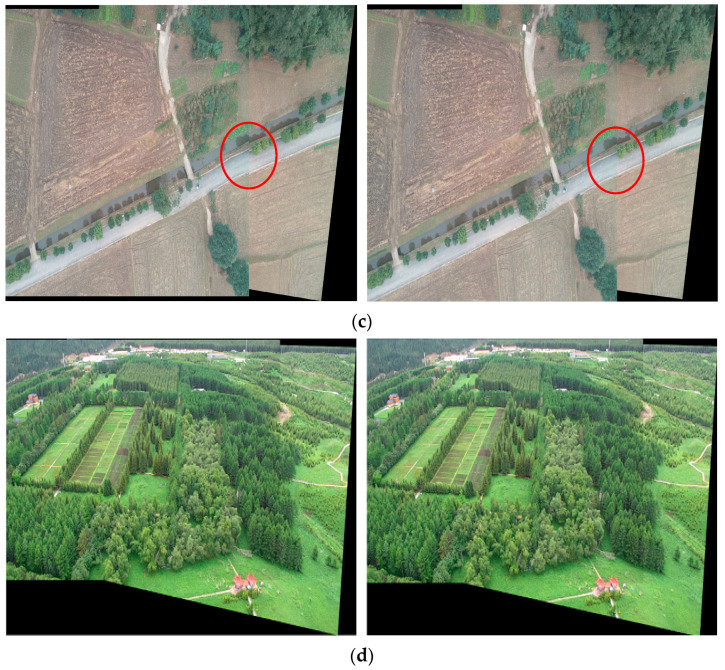
The stitched images of four scenes by the proposed registration algorithms. The left one is obtained according to the first bi-directional matching strategy, and the right one is generated by using the second bi-directional matching strategy: (**a**) roads; (**b**) buildings; (**c**) farmlands; (**d**) forests. Some obvious mismatches are marked by red circles.

The results of bi-directional matching for the other four scene image pairs are in Figure 10, and the final stitched images are displayed in Figure 11. It can be observed from Figure 11a that in the road scene, there are slight mismatches for one road in the mosaicking result of the first bi-directional matching strategy. The reason may be that the less matched point is extracted by this strategy. In Figure 11b, there are some obvious boundary effects in the body of a transmission tower, which may be due to its incomplete appearance at the edge of the reference image. From Figure 11c,d, it can be thought that though there are no significant edge features such as points and lines in the scenes of farmland and forest, the proposed fast registration provides good results for these image pairs. It can be noted that only a visible mismatch for one road exists in the result of the first bi-directional matching strategy for the farmland scene. This may be owing to its insufficient matched point pairs, as shown in Figure 10c. However, some areas for road and farmland scenes appear blurred owing to the quality of input images. Specifically, the red edge images of the farmland scene regarded as blue components in registration are inherently not clear enough. In addition, imaging conditions, such as shooting heights, sensor characteristics, atmospheric visibility and wind speed, light intensity, and the stability of drone platforms, may all affect the quality of drone images.

## 4. Conclusions

Since the ORB algorithm is not able to balance accuracy and time in the registration of UAV-visible images, in this work, a fast automatic registration method for UAV images is proposed by combining the ORB operator with bi-directional matching strategies. The KNN-based search method and a similarity measurement are assembled in initial matching. Two bidirectional matching strategies are designed for fine matching, and the PROSAC algorithm is employed to remove mismatches. In order to verify its performance, the proposed algorithm is compared with the existing fast algorithm for the registration of UAV images from different sensors and scenes. From the experimental results, it can be thought that the improvements of this work are effective. The proposed registration algorithm can enhance matching accuracy for UAV images without requiring any training or prior knowledge while maintaining the high registration speeds of the original ORB algorithm. The maximum accuracy improvement for the experimental images reaches 25.95% for the building scene. In terms of processing time and accuracy, the bi-directional matching method of reducing matched point pairs slightly outperforms the method of increasing matched point pairs. In addition, the proposed registration algorithm needs no training or prior knowledge with low complexities, which is quite suitable for onboard realization.

## Figures and Tables

**Table 1:** The sources and parameters of UAV images used in the registration experiments.

Scenes	Sensors	Image Resolution	Plots	Shooting Time	Shooting Height (m)	Focal Length (mm)
urban	Hasselblad H3DII-50	2044 × 1533	Toronto, Canada	June 2014	500	80
road	Canon IXUS 220HS	2000 × 1500	Brig, Switzerland	September 2013	200	4
building	SONY NEX-7	3000 × 2000	Dortmund, Germany	June 2014	50	16
farmland	Parrot Sequoia	2404 × 1728	Dayi, China	September 2017	80	5
forest	ZENMUSE Z30	1920 × 1080	Wusu Foshan Forest Park, China	June 2019	152	10

**Table 2:** RMSE comparison of registration accuracy of different algorithms (pixels).

	Methods	SIFT	SURF	KAZE	AKAZE	ORB	Ours (Strategy 1)	Ours (Strategy 2)
Scenes								
urban		1.2322	1.1242	1.1846	1.0206	1.3282	**0.9926**	1.0750
road		1.1805	1.2670	1.2945	1.1546	1.3530	**1.0124**	1.1757
building		1.1758	1.2918	1.2173	1.1163	1.3273	**0.9828**	1.0471
farmland		1.3735	1.3905	1.2668	1.1606	1.2005	1.0853	**0.9909**
forest		1.2692	1.3344	1.2287	1.2327	1.2871	**1.0490**	1.0776

The best results for different scene are bolded.

**Table 3:** Time consumption comparison of different registration algorithms (s).

	Methods	SIFT	SURF	KAZE	AKAZE	ORB	Ours (Strategy 1)	Ours (Strategy 2)
Scenes								
urban		125.254	64.023	128.408	101.053	2.252	3.922	4.031
road		137.025	86.799	180.031	144.146	2.324	3.483	3.572
building		163.348	139.725	194.028	117.054	3.457	4.948	4.992
farmland		50.899	46.652	72.712	35.768	1.414	3.562	3.775
forest		93.889	42.794	84.774	103.447	1.727	3.315	3.295

## Data Availability

The source codes involved in this work are available from the authors upon reasonable request.
